# Fiber-Reinforced Polymer Laminates in Aviation and Structural Engineering: A Synthetic Comparison of Performance Requirements, Design Principles, and Defect Assessment Procedures

**DOI:** 10.3390/ma18214938

**Published:** 2025-10-29

**Authors:** Joana Janeikaitė, Ieva Misiūnaitė, Viktor Gribniak

**Affiliations:** 1 Antanas Gustaitis’ Aviation Institute, Vilnius Gediminas Technical University, Linkmenų Street 28-4, 08217 Vilnius, Lithuania; joana.janeikaite@gmail.com; 2 Laboratory of Innovative Building Structures, Vilnius Gediminas Technical University (VILNIUS TECH), Saulėtiekio Avenue 11, 10223 Vilnius, Lithuania; ieva.misiunaite@vilniustech.lt

**Keywords:** fiber-reinforced polymer laminates, damage-tolerant design, durability-based design, fatigue, environmental degradation, non-destructive testing, structural health monitoring, service life prediction, progressive damage modeling

## Abstract

Fiber-reinforced polymer (FRP) laminates are widely used in both aviation and structural engineering, yet their implementation reflects fundamentally different paradigms. Aviation represents a fatigue-critical, certification-driven domain, while structural engineering emphasizes long-term durability and environmental resilience. These sectors were selected as conceptual extremes to explore how contrasting design philosophies, degradation mechanisms, and inspection strategies shape the performance and reliability of laminated FRP composites. Their approaches offer complementary insights: aviation contributes high-fidelity modeling and embedded monitoring, while structural engineering provides scalable inspection strategies and exposure-based degradation logic. Both sectors employ classical laminate theory and finite element modeling, but diverge in modeling depth and regulatory integration. This review synthesizes these contrasts based on 168 literature references, including 141 published between 2020 and 2025, reflecting recent developments in composite design, modeling, and inspection. It contributes to materials engineering by proposing hybrid modeling and inspection frameworks that integrate progressive damage simulation with durability-based design logic. By bridging the modeling precision of aviation with the environmental realism of structural engineering, this review outlines a pathway toward unified, sustainable, and adaptive engineering practices for laminated FRP composites.

## 1. Introduction

Fiber-reinforced polymer (FRP) composites have become indispensable in both aviation and structural engineering due to their high strength-to-weight ratio, corrosion resistance, and adaptability to complex geometries. These materials are increasingly used in primary and secondary load-bearing components, offering performance advantages over traditional materials such as steel and aluminum. However, the implementation of FRP laminates in aviation and structural engineering reflects two fundamentally different paradigms. Aviation represents a fatigue-critical and certification-driven domain, where composite structures must tolerate damage while maintaining predictable residual strength under cyclic and impact loading [1,2]. In contrast, structural engineering operates within a framework that emphasizes durability, cost sensitivity, and long-term environmental resistance, as well as serviceability and economic feasibility [3,4]. This contrast positions the two sectors as conceptual extremes in FRP laminate application, making them feasible for comparative analysis. By examining their respective design philosophies, degradation mechanisms, and modeling strategies, this review aims to identify transferable methodologies and foster cross-sectoral learning that can enhance composite performance across diverse engineering contexts.

This review aims to systematically compare the use of laminated FRP composites in aviation and structural engineering, highlighting how sector-specific demands shape material architecture, degradation mechanisms, modeling approaches, and inspection strategies, developing a synthetic view of the considered aspects. The review adopts a targeted narrative format rather than a systematic protocol. The aim is to synthesize sector-specific mechanisms and modeling philosophies rather than to compile an exhaustive evidence inventory. References were selected to support comparative analysis across aviation and structural engineering, with emphasis on recent developments and methodological contrasts. The focus on laminated configurations—excluding pultruded profiles and other composite systems—is deliberate. Laminated FRPs offer greater flexibility in tailoring mechanical performance through stacking sequences and ply orientation, making them central to both fatigue-critical aerospace structures and durability-driven civil infrastructure [5,6]. As literature reflects [7,8,9], pultruded profiles, while relevant in structural engineering, are typically standardized and less representative of the modeling and degradation complexities addressed in this review. Moreover, other FRP systems, such as textile-reinforced or sandwich composites, though important, fall outside the scope of this comparative analysis due to their distinct structural behavior and application domains [10,11,12].

The manuscript is structured to reflect the comparative logic of the review and to facilitate a coherent analysis of laminated FRP composites in aviation and structural engineering. Section 2 introduces the architectural principles of laminated composites, focusing on ply orientation, stacking sequences, and constituent materials. It compares how aviation and structural engineering prioritize different design constraints—such as weight optimization, damage tolerance, and environmental durability—and introduces classical laminate theory (CLT) as a shared analytical foundation. This section also outlines how laminate configuration influences failure mechanisms and regulatory compliance, establishing the basis for sector-specific degradation pathways.

Section 3 and Section 4 jointly examine the operational and deterioration contexts that shape the performance and reliability of FRP laminates. Section 3 analyzes service-specific loading conditions and environmental stressors, contrasting fatigue-critical aviation environments with the long-term exposure scenarios typical of civil infrastructure. It also discusses how these conditions influence damage evolution, safety philosophies, and service life prediction. Section 4 builds upon this by evaluating degradation mechanisms and damage detection strategies, including matrix cracking, delamination, and fiber–matrix debonding. It compares the implementation of non-destructive testing (NDT) and structural health monitoring (SHM) techniques, highlighting the divergence in inspection resolution, frequency, and integration across sectors. Together, these sections characterize the operational logic and inspection philosophies that underpin sector-specific maintenance regimes.

Section 5 focuses on predictive modeling and degradation assessment, contrasting the empirical reduction approaches commonly used in structural engineering with the progressive damage models and multiscale simulations employed in aviation. It discusses how CLT and finite element modeling (FEM) are extended or constrained depending on sectoral priorities, and introduces hybrid modeling frameworks that integrate physics-based and data-driven techniques. The section also evaluates calibration practices, validation standards, and regulatory constraints, emphasizing the role of modeling depth and data resolution in shaping simulation fidelity.

Section 6 synthesizes the comparative insights developed in Section 2, Section 3, Section 4 and Section 5 and evaluates their implications for cross-sectoral integration. It discusses how the precision of aviation modeling and the durability logic of structural engineering can be combined to develop more resilient and certifiable composite structures. The section also verifies the alignment of the manuscript’s conclusions with recent literature and identifies opportunities for methodological advancement.

The final section concludes the review by summarizing key findings and outlining future research directions aimed at unifying modeling and inspection methodologies for laminated FRP composites. The comparative dimensions addressed in Section 2, Section 3, Section 4 and Section 5 are synthesized in Table 1, which serves as a methodological anchor for the review. It summarizes the sector-specific contrasts in FRP laminate design, degradation, modeling, and inspection, providing a structured lens through which the manuscript’s contribution to materials engineering can be understood.

The results summarized in Table 1 reveal a fundamental divergence in how FRP laminates are designed, implemented, and maintained across the considered sectors. Aviation engineering emphasizes lightweight optimization, fatigue resistance, and damage tolerance, supported by multiscale modeling and embedded monitoring systems. Structural engineering, in contrast, prioritizes robustness, environmental durability, and cost-effective inspection, often relying on simplified modeling and empirical degradation factors. These differences reflect not only distinct operational demands but also divergent regulatory frameworks and safety philosophies.

This divergence has led to a fragmentation of engineering knowledge, where aviation and structural engineering have developed specialized but isolated methodologies for modeling, inspection, and degradation assessment of laminated FRP composites. Aviation emphasizes fatigue-critical performance and certification-driven modeling, while structural engineering prioritizes environmental durability and scalable inspection. The lack of integration between these approaches limits the broader applicability of sector-specific innovations. A unified understanding of FRP laminate behavior—one that bridges fatigue-tolerant and durability-driven paradigms—is essential for advancing composite design, service life prediction, and maintenance strategies across engineering domains.

This systematic review addresses the gap by comparing the design philosophies, degradation mechanisms, modeling strategies, and inspection practices of laminated FRP composites in aviation and structural engineering. It identifies transferable methodologies and highlights opportunities for integrating high-fidelity damage modeling with durability-based design logic. Thus, the manuscript contributes to materials engineering by proposing a hybrid perspective that leverages the strengths of both sectors. This perspective supports the development of unified modeling and inspection frameworks that are not only certifiable and resilient but also scalable and environmentally robust. The comparative analysis substantiates the selection of aviation and structural engineering as conceptual extremes. It demonstrates how their diverse approaches can reinforce each other to ensure the sustainable and progressive development of FRP composite technologies.

## 2. Design of Components and Laminate Architectures

This section examines the architectural principles and design constraints governing laminated FRP composites in the aviation and structural engineering industries. It explores how sector-specific performance demands, safety philosophies, and environmental conditions shape laminate configurations, deterioration mechanisms, and modeling practices. The discussion is structured into four subsections: a comparative overview of design logic and failure mechanisms, followed by detailed analyses of aviation and structural engineering strategies, and concluding with a synthesis of regulatory frameworks.

### 2.1. Comparative Design Logic and Deterioration Mechanisms in Laminated FRP Composites

The design of laminated FRP composites is shaped by the operational demands and safety philosophies of the sectors in which they are deployed. In aviation, laminate architecture is optimized for weight reduction, fatigue resistance, and damage tolerance, often under stringent certification constraints. Structural engineering, in contrast, emphasizes long-term durability, cost-efficiency, and resistance to environmental degradation, with design decisions governed by serviceability and life-cycle performance.

As discussed in Section 1, both sectors rely on ply orientation, stacking sequences, and constituent material selection to tailor mechanical behavior. However, the prioritization of design objectives—such as fail safety in aerospace versus redundancy in infrastructure—leads to markedly different laminate configurations and deterioration pathways. Numerous studies have documented how thin-ply architectures suppress early-stage damage in aerospace applications [13,14,15,16]. In contrast, structural FRPs often exhibit externally visible degradation due to moisture ingress, ultraviolet (UV) exposure, and freezing–thawing cycles [17,18,19].

To synthesize these observations, Table 2 presents a comparative summary of the dominant failure mechanisms encountered in laminated FRP composites across aviation [15,20,21,22,23,24,25,26,27,28,29,30] and structural engineering [7,17,18,19,31,32,33,34,35,36]. This table classifies each failure manner by sector, outlines its consequences, and identifies suitable detection methods. This structured overview provides a foundation for understanding how design philosophies are shaped by anticipated deterioration risks and inspection capabilities.

The insights from Table 2 emphasize the need for sector-specific design strategies. In aviation, hidden damages such as delamination and matrix cracking require embedded monitoring and conservative safety margins. In structural engineering, visible degradation and environmental aging lead to the adoption of simplified layups and empirical reduction models. These differences will be further explored in the following subsections, which detail the architectural principles and design constraints unique to each area. The comparison in Table 2 illustrates how the type of damage impacts the destruction of material and how damage is controlled, depending on the specific application within a particular industry. The upcoming sections analyze how these mechanisms are addressed through architectural and structural engineering strategies.

### 2.2. Aviation: Optimized Layups, Thin Plies, and Damage-Tolerant Design

In aviation structures, laminated FRP composites are designed to meet stringent performance and certification requirements [38]. The architectural configuration of these laminates—defined by ply orientation, stacking sequence, and constituent materials—is optimized to balance stiffness-to-weight efficiency, fatigue resistance, and damage tolerance.

Conventional quasi-isotropic layups (e.g., [0°/±45°/90°]) distribute plies in multiple directions to achieve balanced in-plane stiffness and minimize warping and residual thermal stresses [15,39]. Thus, 0° plies enhance axial stiffness, ±45° plies provide shear and torsional resistance, and 90° plies contribute to transverse rigidity and thermal stability. More advanced stacking configurations—such as those proposed in Tsai’s “Double-Double” laminate concept—employ tailored ply orientations (e.g., [±*φ*, ±*ψ*]) to reduce bending–twisting coupling and enhance weight efficiency without compromising structural robustness [40,41]. In this context, *φ* and *ψ* denote optimized fiber angles selected to balance in-plane stiffness and minimize coupling effects. Investigations [30,31,42,43] have demonstrated that such modifications to the stacking sequence significantly affect damage resistance, fatigue performance, and impact tolerance in aerospace-grade laminates.

Recent innovations include gradient ply thickness distributions, which mitigate interlaminar stresses and enhance low-velocity impact resistance [43], and hybrid thin-thick architectures, which combine the stiffness of thick plies with the crack-arresting benefits of thin plies [42]. These strategies reflect a shift toward tailoring laminate architecture to suppress damage initiation and delay its progression under complex loading scenarios.

A key advancement in aerospace composites is thin-ply technology, which employs plies thinner than 100 μm to suppress early matrix cracking and delay delamination. Damage-tolerant architectures such as thin-ply designs and hybrid stacking sequences have demonstrated superior fatigue resistance and improved compression-after-impact (CAI) performance across diverse loading conditions [13,14,15,21,44,45,46,47]. Their ability to confine damage to localized regions enhances tolerance to BVID, a critical concern in airframe design. Studies [48,49] show that BVID can reduce residual strength by up to 60% despite minimal surface evidence. In contrast, thin-ply laminates increase delamination thresholds and absorb more impact energy depending on fiber orientation and impact direction [14,16]. Thus, developing the damage-tolerant structures minimizes reliance on conservative safety margins and requires extensive experimental validation.

Manufacturing FRP composites remains a challenge: autoclave curing ensures aerospace-grade quality but is costly and limits scalability. While out-of-autoclave (OOA) processes offer sustainability advantages, they are not yet fully certified for primary structural components [50].

Damage-tolerant design is a cornerstone of aviation laminate philosophy [51]. Structures must remain safe and predictable even when flaws are present but undetected. This target is achieved through the use of toughened resin systems, interleaving strategies, and optimized stacking sequences that arrest crack growth and stabilize delamination [13]. Material selection—particularly resin chemistry and interleaf composition—directly influences fiber–matrix adhesion, crack initiation thresholds, and environmental resistance [21,27].

Failure mechanisms such as fiber kinking under compression and inter-fiber (matrix-dominated) fractures are mitigated through thin-ply architectures and progressive modeling [21,27]. While CLT provides baseline stiffness estimates, aviation design increasingly relies on progressive damage models to simulate complex interactions among matrix cracking, fiber breakage, and delamination growth [52]. To clarify the role of thin-ply configurations, Table 3 summarizes common product forms, nominal ply thicknesses, and their influence on delamination thresholds, fatigue slope, and CAI retention. This table highlights how thin-ply architectures suppress early damage and enhance post-impact performance through mechanisms such as crack arrest, stress redistribution, and delayed delamination onset. These trends support the adoption of thin-ply designs in fatigue-critical aerospace applications, where localized damage must remain stable under cyclic loading. The improved CAI retention and flatter fatigue slopes observed in thin-ply and hybrid stacks contribute to the damage-tolerant philosophy discussed in Section 2.1.

Despite these innovations, certification authorities, including the Federal Aviation Administration (FAA) and the European Union Aviation Safety Agency (EASA), impose conservative knock-down factors to account for inspection limitations and hidden damage, partially offsetting the weight savings achieved by optimized designs [53,54,55,56]. Environmental durability further constrains long-term performance, as moisture ingress, UV exposure, and thermal cycling degrade the fiber–matrix interfaces, accelerating fatigue and delamination [13,28,29].

Taken together, aviation laminate design reflects a multi-constraint optimization problem. Performance must be balanced against damage tolerance, environmental resistance, manufacturability, and certification safety margins. The resulting design philosophy extends beyond stiffness-to-weight optimization, emphasizing fail safety, inspectability, and long-term reliability as defining features of aerospace composites [1,57]. While aviation laminate design is driven by fatigue-critical performance and certification constraints, structural engineering adopts a fundamentally different approach [28,30]. The following section examines how durability, cost efficiency, and environmental exposure influence laminate configurations in civil infrastructure.

### 2.3. Structural Engineering: Simplified Layups and Durability-Driven Design

In structural engineering, laminated FRP composites are increasingly used in bridges, buildings, and retrofitting applications [58,59]. Unlike aviation, where design optimization is driven by weight sensitivity and certification constraints, structural FRP design is governed by long-term durability, serviceability, and cost efficiency [3,4]. These priorities shape laminate architecture, stacking sequences, and material selection, resulting in configurations that emphasize robustness and environmental resistance over weight minimization.

Laminate configurations in structural engineering typically employ unidirectional or quasi-isotropic layups optimized for bending and shear performance under static or sustained loads [7,60,61]. The simplicity of these stacking sequences ensures manufacturability and reproducibility, which are essential for large-scale deployment. Hybrid laminates combining glass and carbon fibers are increasingly adopted to balance stiffness, cost, and environmental resilience [62,63,64,65,66].

Unlike aerospace composites, which often fail due to hidden damage (Table 2), structural FRPs exhibit externally visible degradation mechanisms. These damaging processes include surface cracking, fiber–matrix debonding, interlaminar shear failure, and adhesive delamination at bonded interfaces. As summarized in Table 2, such deterioration is typically driven by environmental exposure, including moisture ingress, ultraviolet radiation, freezing–thawing cycles, and chemical attack, which progressively weaken the fiber–matrix interface and reduce bond capacity [17,18,19,37].

Matrix cracking and surface whitening often serve as early indicators of damage [17,35,37]. At the same time, long-term creep and fatigue under sustained loading can lead to stiffness loss and excessive deflections, particularly in bridges and large-span structures [17,18,37]. Hybrid laminates are particularly susceptible to mismatch-driven failures, where differences in thermal expansion or stiffness between fiber types intensify interlaminar stresses and accelerate degradation [17,18].

From a life-cycle cost (LCC) perspective, FRP laminates offer substantial long-term savings despite higher initial material costs. Their corrosion resistance and reduced maintenance requirements contribute to extended service life and lower environmental impact [63,67]. As a result, structural FRP design emphasizes redundancy, inspectability, and resilience to harsh environmental conditions rather than weight optimization [68].

Design decisions are further constrained by the limitations of visual inspection and the absence of embedded monitoring systems [17,32]. Periodic or reactive inspection regimes dominate field practice, relying on thermography, acoustic emission, and manual surveys to detect damage [31,37]. This practice stands in contrast to aviation, where embedded sensors and scheduled NDT are integral to certification workflows (Table 2).

Overall, structural engineering adopts a pragmatic design philosophy. Laminate architectures are deliberately simplified to ensure manufacturability and predictable load transfer [69]. Durability, serviceability, and inspection feasibility govern design logic, reinforcing environmental resistance as the defining dimension of performance in civil infrastructure. These durability-driven design strategies are further constrained by regulatory frameworks that define allowable stresses, exposure classifications, and inspection requirements. Section 2.4 provides a comparative overview of the standards and certification practices that govern laminate design in both sectors.

### 2.4. Standards, Codes, and Certification Frameworks for FRP Laminates

The design of laminated FRP composites is not solely governed by mechanical performance or environmental durability—it is also shaped by the regulatory frameworks and certification standards that define allowable stress levels, inspection intervals, and modeling fidelity. These frameworks differ substantially between aviation and structural engineering, reflecting the distinct safety philosophies and operational contexts of each sector.

In aviation, composite structures are subject to rigorous certification procedures. Regulatory bodies, such as FAA and EASA, mandate damage-tolerant design and require that residual strength be maintained, even in the presence of flaws like delamination or BVID [49]. The FAA [53] and EASA [56] regulations outline specific requirements for structural integrity, inspection protocols, and modeling validation. The handbook [54,55] supports these principles, which develop standardized procedures for material characterization, building-block testing, and simulation workflows. Aviation modeling practices are deeply embedded in the certification process. CLT forms the analytical foundation, but it is routinely extended through the use of progressive damage models, cohesive zone elements, and multiscale simulations [70,71]. These models are calibrated using ply-level mechanical and fatigue data, validated through coupon, element, and subcomponent testing. The emphasis on high-fidelity prediction reflects the sector’s reliance on simulation to anticipate damage evolution and ensure airworthiness.

In contrast, structural engineering operates within a durability-based design paradigm [6]. Regulatory documents emphasize long-term serviceability and environmental resistance rather than damage tolerance. The *fib* Bulletin [72], ACI Committee 440 guideline [73], and the National Research Council guideline [74] guide the use of externally bonded FRP systems for strengthening concrete structures, including exposure classifications and reduction factors for environmental degradation. The European standard (Eurocode 2) [75] introduces provisions for FRP reinforcement in concrete design, although its scope remains limited and primarily focused on bar-type composites. Model Code 2020 [76] also presents the FRP reinforcement principle, albeit in a general manner. The fragmented regulation of FRP composites in structural engineering stems from the absence of standard rules for the material properties and mechanical performance of FRPs, whose properties vary substantially in many instances [77].

Design codes in structural engineering typically rely on empirical degradation models [5]. Environmental reduction coefficients—derived from accelerated aging tests—are applied to tensile strength and strain limits to account for moisture ingress, UV exposure, and freezing–thawing cycles. For example, the technical report [78] recommends a tensile strength reduction factor of approximately 0.51 for carbon fiber-reinforced polymer (CFRP) laminates exposed to harsh conditions. At the same time, the design guideline [74] prescribes exposure-dependent strain reduction coefficients ranging from 0.85 to 0.50.

To synthesize these regulatory distinctions [53,54,55,56,72,73,74,75,76,78,79,80,81,82,83,84,85,86,87,88,89,90], Table 4 presents a comparative summary of the principal standards and guidelines governing laminated FRP design in aviation and structural engineering. It highlights the scope of each document, its role in modeling and certification, and its relevance to laminate-specific degradation mechanisms. Citations are provided at the document level. FAA and EASA airworthiness rules (e.g., CS-25) [53,56] are binding through the certification basis, whereas advisory circulars (ACs) and acceptable means of compliance (AMCs) serve as non-binding guidance. In civil engineering, provisions in ACI 440, *fib* Bulletins, CNR, and Eurocode documents are normative only when adopted by the relevant authority or owner; otherwise, they function as recommended practice.

The comparison in Table 4 reveals a fundamental divergence in how regulatory frameworks influence laminate design. Aviation standards prioritize predictive modeling and damage-tolerant certification, while structural engineering codes emphasize empirical degradation factors and conservative safety margins. These differences reflect not only distinct operational risks but also the maturity of modeling practices and the availability of high-resolution data in each sector. Looking forward, harmonizing these approaches—by integrating high-fidelity modeling with durability-based design logic—may enable more resilient and certifiable composite structures across industries.

## 3. In-Service Conditions and Environmental Effects

The service performance of FRP laminates is governed not only by their intrinsic material properties but also, and more decisively, by the conditions to which they are exposed during operation. Across application domains, these conditions encompass a spectrum of mechanical loadings and environmental stressors that act singly or in combination to initiate and propagate damage. In aviation, high-frequency cyclic pressurization, vibratory loads, and transient impacts interact with thermal excursions and intermittent moisture exposure to shape the progression of fatigue and impact-related degradation. In structural infrastructure, in contrast, sustained static loads and low-amplitude cyclic actions are coupled with prolonged exposure to humidity, ultraviolet radiation, freezing–thawing cycles, and chemical agents, resulting in gradual deterioration over extended timescales.

Such operational contrasts underline that the governing degradation mechanisms, inspection practices, and design philosophies cannot be considered in isolation from the service environment. Instead, it is the interplay between loading spectra and environmental stressors that dictates how damage initiates and evolves, ultimately shaping maintenance strategies, safety margins, and regulatory frameworks across different sectors.

### 3.1. Mechanical Loading Spectra

Across operational domains, FRP laminates experience markedly different loading regimes in terms of frequency content, magnitude, and duration. In aviation, structures are exposed to high-frequency cyclic loads originating from repeated fuselage pressurization and depressurization cycles, aerodynamic gust responses, and maneuver load sequences during typical missions. These load cycles—often numbering in the tens of thousands over an airframe’s service life—are characterized by relatively high stress amplitudes but short load durations, making fatigue the dominant life-limiting mechanism in the absence of significant impact damage [21,91]. The damage tolerance philosophy embedded in the FAA and EASA regulations, therefore, requires that laminates maintain residual strength above the ultimate load capability even after detectable impact events.

Impact loading in aviation encompasses bird strikes, hailstone impacts, runway debris, and dropped tooling, each of which can initiate damage mechanisms, as summarized in Table 2, including matrix cracking, fiber fracture, and delamination, within milliseconds. Studies on composite laminates, including fuselage skins, have shown that impact resistance is strongly influenced by the laminate stacking sequence/fiber orientation, as well as local curvature [92,93]. In parallel, vibratory loads from engine imbalance, rotor-fuselage interactions, and aeroelastic phenomena such as flutter can contribute to fatigue accumulation, particularly in secondary structures. The strategic use of aeroelastic tailoring—exploiting laminate anisotropy to alter stiffness distribution—has emerged as a means to mitigate such vibratory stresses [94,95].

In structural engineering, encompassing civil and military infrastructure, FRP laminates and externally bonded reinforcement systems are predominantly subjected to static and sustained loads derived from self-weight, occupancy, and permanent superimposed actions. These prolonged loadings promote creep and creep-rupture, particularly in polymer matrices under service temperatures approaching the glass transition [34]. Although cyclic fatigue is less prevalent than in aerospace, it can occur in long-span bridges, marine fenders, and defense barriers under traffic-induced, wave-induced, or wind-induced cyclic loading. Field and laboratory research have shown that CFRP-strengthened reinforced concrete elements can exhibit significant reductions in fatigue life when pre-cracked or exposed to aggressive environmental conditions, with dominant failure mechanisms—such as FRP-substrate debonding—outlined in Table 2.

Occasional extreme events—such as earthquakes, windstorms, blast loading, or vehicular impacts—introduce short-duration, high-intensity loads into structural engineering scenarios, producing localized damage patterns that are analogous in some respects to those caused by low-velocity impact in aviation. However, the frequency of such events is much lower, and their incorporation into design spectra is typically addressed via probabilistic hazard modeling rather than continuous-cycle fatigue analysis [96].

Although both the aviation and structural engineering sectors extensively use FRP laminates, their service loading spectra differ markedly. In aviation, composite skins are subjected to hundreds of pressurization cycles annually, each imposing high stress amplitudes—often 30–50% of ultimate laminate strength—from maneuver and gust actions. At the same time, low-velocity impacts such as bird strikes or hail can induce severe localized damage that must be tolerated throughout the service life [24,25,97]. Structural engineering laminates, in contrast, typically experience millions of low-amplitude cycles from traffic or vibrational loads over decades, with stress levels mostly below 10–15% of ultimate strength, resulting in gradual stiffness reduction and micro-damage accumulation rather than abrupt failure [98,99]. This contrast between low-frequency, high-stress loading in aviation and high-frequency, low-stress loading in structural applications underscores the fundamentally different fatigue and damage accumulation patterns observed in the two sectors.

### 3.2. Environmental Stressors

Environmental stressors—such as moisture, UV radiation, and thermal cycling—play a decisive role in laminate degradation, with sector-specific exposure regimes shaping their impact. As discussed in the previous section, the mechanical loading environment is a key factor in the long-term integrity of composite laminates; however, service performance is equally shaped by the interaction of these loads with environmental stressors. In both aviation and structural engineering applications, moisture ingress, UV radiation, and thermal cycling are the principal agents of degradation. However, their range, frequency, and combined severity differ significantly between the two sectors.

Moisture penetration into fiber-reinforced polymer laminates typically follows Fickian diffusion, a process in which the moisture uptake rate is initially proportional to the square root of time before reaching an equilibrium state. Fick’s law of diffusion governs this behavior, assuming constant diffusivity and uniform material properties. In practice, however, environmental cycling, resin plasticization, and microcracking can lead to non-Fickian moisture transport, where deviations from the idealized uptake curve occur due to time-dependent changes in the polymer network, fiber–matrix interfacial degradation, or damage-induced permeability [100,101,102,103]. The absorbed moisture can plasticize the resin, cause swelling, and progressively weaken the interfacial bond between fibers and matrix, leading to measurable reductions in stiffness and strength. Experimental studies indicate that long-term hygrothermal exposure can cause 20–50% losses in tensile or flexural properties, with the extent of degradation strongly dependent on fiber architecture, resin chemistry, and the presence of protective barriers or surface coatings [100,101,102,104].

Although both aviation and structural engineering composites are subject to humidity variations, their exposure regimes differ markedly. Aviation composites typically face intermittent moisture exposure—for example, during ground operations in humid climates or from precipitation—combined with rapid temperature swings between high-altitude flight and ground-level conditions, sometimes multiple times daily. In contrast, structural engineering applications may endure continuous exposure to ambient humidity, rainfall, or even immersion over decades, often under sustained or cyclic loads, which can accelerate diffusion-driven degradation mechanisms [103,104].

Prolonged UV exposure induces chain scission and oxidative reactions in the polymer matrix, particularly in glass- and carbon-fiber composites without UV-resistant surface treatments. Such photo-degradation alters surface morphology, increases brittleness, and diminishes interlaminar toughness. Recent studies on FRP laminates [105,106] report substantial reductions in the mechanical performance after extended UV irradiation, with implications for both primary load-bearing members and secondary structures. In aviation, UV damage is less severe due to coatings and reduced direct exposure; in structural engineering, it remains a dominant degradation mechanism for exterior components.

Thermal cycling is a critical environmental stressor in FRP laminates, primarily due to the mismatch in coefficients of thermal expansion (CTE) between stiff fibers and a comparatively compliant polymer matrix. Repeated thermal excursions—such as transitions from hot ground conditions to high-altitude cold—induce matrix microcracking, which in turn facilitates moisture ingress and accelerates long-term degradation. Cold-temperature cycling studies on carbon/polyetherimide composites have confirmed deterioration in tensile properties after repeated exposure to cryogenic temperatures and ambient conditions [107]. Similar investigations on cured carbon/epoxy laminates have documented the progressive development of microcracking under cycles ranging from −55 °C to +120 °C, with damage accumulation characterized via acoustic emission and X-ray computed tomography [108]. More recent coupled thermo-hygro-mechanical analyses highlight that such thermal fluctuations can interact with moisture ingress to exacerbate stiffness and strength losses, underscoring the importance of multi-physics modeling for reliable service life prediction [109]. In addition, hygrothermal ageing studies on aerospace-grade prepreg-based CFRPs reveal that combined temperature and humidity cycling accelerates resin oxidation and chain scission, further weakening fiber–matrix adhesion [20].

In aviation service, components can experience daily thermal swings from ground temperatures up to +40 °C to cruise-level conditions of approximately −60 °C, resulting in more than 700 high-amplitude cycles annually under typical short-haul operating schedules [110]. In contrast, structural engineering laminates are more often subjected to lower-amplitude but seasonally repetitive variations—typically ranging from −20 °C to +40 °C—with annual freezing–thawing cycles between 50 and 150, depending on the severity of the climate [111].

The most severe degradation often results from the interaction of multiple environmental factors. Synergistic effects—such as those arising from combined UV exposure, thermal cycling, and moisture ingress—have been shown to produce non-linear acceleration of stiffness loss and crack propagation rates. This interplay is especially critical in structural engineering applications, where continuous exposure to multiple stressors is usual. It also warrants consideration in aviation for components operating in humid, high-temperature, or chemically aggressive environments. For instance, recent studies on carbon/epoxy laminates subjected to elevated temperatures and chemical agents (e.g., 10% NaOH, H_2_SO_4_, NaCl) show reductions in tensile performance by up to 35%, along with surface degradation and lowering of glass transition temperatures—clear indicators of accelerated material deterioration [101,102,103,104].

These sector-specific exposure regimes, when coupled with the mechanical demands described in Section 3.1, underscore the necessity for predictive models that incorporate realistic environmental cycles, protective strategies, and multi-physics degradation mechanisms. Such integration is essential for accurately forecasting service life, optimizing maintenance intervals, and ensuring safety margins across both domains.

### 3.3. Implications for Service Life and Safety

The implications of in-service environments for design philosophy become evident when considering how degradation risks translate into safety management strategies. In aviation, the expectation of unavoidable damage under cyclic and impact-dominated conditions has led to the institutionalization of a damage-tolerant approach, in which residual strength and inspection intervals are explicitly linked to defect growth and repair thresholds. Structural engineering practice, in contrast, addresses environmental exposure through a durability-based, predominantly safe-life philosophy, embedding reduction factors and conservative partial safety margins into design codes to mitigate long-term property loss without relying on frequent inspection. These divergent frameworks illustrate how the anticipated influence of environmental conditions on laminate degradation directly informs the choice of service life philosophy in each sector.

In aviation, two philosophies have historically guided structural safety: the safe-life approach and the damage-tolerant approach. The safe-life method, dominant during the early introduction of composite structures, required that all primary components be designed to withstand the entire service life without detectable damage or significant property loss. While this approach ensured high reliability, it often resulted in conservative design margins and the premature retirement of otherwise serviceable structures [21,22,24]. In modern airframe design, the emphasis has shifted towards a damage-tolerant philosophy, mandated by regulatory bodies such as the FAA and EASA. Under this framework, structures are explicitly required to sustain service loads even in the presence of damage mechanisms such as fatigue cracks, delamination, or BVID (Table 2). Safety is thereby assured through a combination of robust residual strength and systematic inspection intervals, calibrated against the anticipated growth of defects under cyclic pressurization, vibratory loading, and environmental degradation [2,22,94,95]. Non-destructive inspection (NDI) methods, such as ultrasonics, thermography, and acoustic emission, play a central role in this regime, as they provide the means to detect and monitor sub-critical defects before they reach thresholds necessitating repair [97,112]. The consequence is a proactive maintenance strategy in which inspection frequency, repair criteria, and safety margins are all tied directly to post-damage behavior, rather than pristine properties [21,112,113].

Structural engineering, in contrast, does not typically operate under a damage-tolerant paradigm. The sheer scale and distributed nature of infrastructure make frequent inspections impractical, and the service lives expected—often exceeding 50 years—demand fundamentally different strategies. Instead, the prevailing philosophy is durability-based and safe-life oriented, in which materials are designed to retain adequate performance throughout their intended lifespan with minimal intervention. This approach is operationalized through environmental reduction factors applied in design codes and guidelines, most notably the ACI guideline [73] and *fib* Bulletin [72], which adjust allowable stresses to account for long-term degradation resulting from hygrothermal aging, freezing–thawing cycles, ultraviolet radiation, or chemical attack [102,104,111,114]. In this framework, safety is assured not by repeated inspection but by incorporating conservative design margins and durability provisions—such as resin-rich layers, protective coatings, or controlled exposure classifications—into the initial design [113,115]. Recent advances have also introduced probabilistic methods and reliability-based calibration of safety factors, allowing for site-specific adaptation to exposure conditions while maintaining code-level conservatism [114,116].

These different approaches demonstrate how environmental stressors impact safety management in distinct ways. In aviation, moisture ingress and thermal cycling can reduce inspection intervals by accelerating fatigue damage; however, structures remain operational under a damage-tolerant framework with specific inspection limits [20,21,112]. In civil infrastructure, these same environmental factors are viewed as gradual, and baseline deterioration is managed through conservative design rather than active monitoring [113,114,115,116]. Therefore, while aviation depends on detect-and-repair strategies within a highly regulated operational setting, structural engineering uses design-in durability strategies that minimize the need for in-service inspections.

The divergence between aviation and civil infrastructure highlights how service life philosophies are inherently shaped by operational context. In the aerospace sector, the prevalence of short but intensive load cycles, combined with the criticality of structural safety, has driven the adoption of damage-tolerant design supported by rigorous inspection and maintenance protocols. In structural engineering, in contrast, the extended service horizons of large-scale infrastructure, coupled with the impracticality of continuous monitoring, necessitate a durability-based approach that embeds conservative safety margins at the design stage. Taken together, these contrasting strategies highlight the pivotal role of the service environment and usage patterns in defining not only the expected degradation trajectories of FRP laminates but also the frameworks by which their long-term performance and reliability are ensured. The degradation mechanisms discussed above—shaped by sector-specific loading regimes and environmental exposure—necessitate the implementation of tailored inspection and monitoring strategies. Section 4 builds upon this foundation by examining how aviation and structural engineering address these challenges through damage detection methodologies and assessment protocols.

## 4. Degradation Mechanisms and Damage Detection

The degradation behavior of FRP laminates is governed not only by their material architecture but also by the duration and severity of service conditions, which differ substantially between aviation and structural engineering applications. In aviation, cyclic and impact loads are the primary concerns, prompting the adoption of damage-tolerant design and high-resolution inspection protocols. Structural engineering, in contrast, emphasizes long-term durability under environmental stressors such as moisture, ultraviolet radiation, and freezing–thawing cycles, with damage detection often relying on periodic or reactive inspection strategies. This section examines how these sector-specific conditions impact the progression of damage, the implementation of NDT and SHM techniques, and the development of inspection regimes tailored to operational requirements.

### 4.1. Comparative Overview of Degradation Mechanisms and Detection Practices

The degradation behavior of FRP laminates reflects the interplay between material architecture and service-specific operational demands, which differ markedly between aviation and structural engineering. These differences have already been outlined in Section 2 and Section 3, particularly in Table 2, which summarizes the dominant failure mechanisms and their sectoral manifestations. Building on that foundation, this section focuses on how these degradation patterns influence damage detection strategies and inspection philosophies.

In aviation, degradation typically initiates at the ply level through matrix cracking and fiber–matrix debonding, which evolve into delamination and fiber fracture under cyclic loading. BVID, caused by low-velocity impacts such as tool drops or hail, is particularly critical due to its subsurface nature and its effects on CAI performance [27,30,39]. Detection of such damage requires embedded SHM systems and scheduled NDT using UT, AE, and thermography [23,117,118].

In structural engineering, degradation is more often externally visible and environmentally driven. Moisture ingress and UV exposure degrade the resin matrix and fiber–matrix interface, leading to surface cracking, adhesive debonding, and interlaminar shear failure [31,37,59,119]. These mechanisms are typically monitored through periodic or reactive inspection regimes, including visual surveys, thermographic scans, and AE-based monitoring. The absence of embedded SHM systems and the scale of infrastructure necessitate pragmatic detection strategies focused on member-level performance [35,37].

To synthesize these sector-specific differences, Table 5 presents a comparative overview of degradation mechanisms and damage detection practices in aviation and structural engineering reported in the references [6,15,17,18,21,22,23,24,26,27,30,31,33,35,36,37,38,39,48,49,53,56,60,76,117,118,119,120,121,122,123,124,125,126]. The table complements the failure mode classification in Table 2 by emphasizing inspection strategies, detection resolution, and operational philosophies.

The distinctions summarized in Table 5—particularly the contrast between fatigue-critical and durability-driven degradation, the difference in inspection resolution and frequency, and the divergence in SHM integration—underscore the need for tailored damage assessment protocols that reflect the operational realities of each sector. While aviation demands high-fidelity, ply-level monitoring embedded within certification workflows, structural engineering relies on scalable, cost-effective inspection tools and empirical degradation models. These protocols have evolved naturally, shaped by sector-specific demands, constraints, and safety philosophies—not due to a lack of awareness of alternative approaches, but as optimized responses to practical needs. In this context, detection resolution refers to the scale at which damage is identified: ply-level monitoring targets individual composite layers (typical in aviation), member-level detection focuses on entire structural components (typical in structural engineering), and surface-level inspection addresses visible or near-surface damage.

To complement the qualitative contrasts presented in Table 5, Table 6 introduces representative quantitative ranges compiled from experimentally reported datasets and handbooks. These values provide order-of-magnitude context for knock-down factors, residual CAI strength after BVID, fatigue slopes, moisture diffusion coefficients, UV-induced strength loss, inspection intervals, and modeling fidelity. These characteristics are indicative of magnitude and trend, and are not suitable for use as certified design limits.

The quantitative indicators in Table 6 are consistent with the sectoral patterns summarized above. Aviation remains impact- and fatigue-critical, driving the need for scheduled NDT and embedded SHM within a building-block validation framework. Structural engineering practice is durability-dominated, shaped by moisture/UV exposure and interface degradation, and relies on program-based inspections and global analyses with empirically derived environmental reductions. Inspection intervals are program-specific in both sectors; the entries reflect inspection philosophy and documented practices, where available, rather than universal values. Modeling fidelity also diverges, with aviation employing progressive damage and multiscale simulations, while civil engineering relies on CLT/FEM with reduction factors.

These comparative insights reinforce the sector-specific optimization paths and highlight the potential for cross-sectoral learning. For instance, the precision of aviation-grade SHM systems could inform the development of scalable monitoring solutions for critical infrastructure. At the same time, the durability logic of structural engineering may offer insights into long-term environmental resistance strategies for aerospace composites. This comparative perspective does not imply convergence of protocols, but rather highlights opportunities for methodological inspiration and selective adaptation, respecting the distinct optimization paths of each field. These implications are further explored in the following subsections and in the modeling discussion of Section 5.

### 4.2. Sector-Specific Damage Evolution and Detection Techniques

Building on the comparative overview in Section 4.1, this subsection examines how sector-specific degradation mechanisms evolve and how they are detected through NDT and SHM techniques. In aviation, degradation typically initiates at the ply level through matrix cracking and fiber–matrix debonding, which evolve into delamination and fiber fracture under cyclic loading [15,23,27]. BVID, often caused by tool drops or hail, is particularly critical due to its subsurface nature and its effects on CAI performance [26,30,39], as discussed in Section 2.2. Thin-ply architectures and optimized stacking sequences are designed to suppress early-stage damage and delay its progression [13,14,21,44,45,47,48,127]. However, environmental stressors such as moisture ingress, UV exposure, and thermal cycling can degrade fiber–matrix interfaces and accelerate fatigue [13,28,29]. These mechanisms necessitate damage-tolerant design and embedded monitoring systems, ensuring that sub-critical damage remains stable until repair thresholds are reached [51,57].

In structural engineering, degradation is primarily driven by long-term environmental exposure and sustained loading. Matrix cracking and surface whitening often serve as early indicators of damage [35,37]. At the same time, moisture ingress, UV radiation, and freezing–thawing cycles degrade the resin matrix and fiber–matrix interface, promoting delamination and adhesive debonding [31,37,60,119]. Interlaminar shear failure is particularly relevant in pultruded and hybrid laminates, where mismatched thermal expansion and stiffness properties exacerbate stress concentrations [17,18]. Unlike aviation, where damage may remain hidden and critical, structural degradation is often externally visible and progresses gradually. Inspection strategies rely on visual surveys, thermography, and AE-based monitoring, with limited use of embedded SHM systems due to cost and scale constraints [37,60].

The sequence and interaction of these degradation mechanisms—from matrix cracking to delamination and fiber fracture—reflect a progressive failure chain that has been extensively characterized under various loading conditions, including chemical, thermal, oxidative, and mechanical stressors [128]. In aviation, this chain is modeled and monitored using embedded sensors and validated through building-block testing. In structural engineering, degradation is typically tracked through member-level performance indicators, such as stiffness loss and deflection trends, which are influenced by environmental exposure and long-term service conditions [129]. To clarify how these degradation mechanisms are detected, Table 7 maps NDT and SHM techniques to specific failure types, indicating their monitoring depth, damage size sensitivity, and typical deployment cadence. Aviation integrates these techniques into scheduled maintenance workflows and certification protocols, forming a cornerstone of the damage-tolerant design philosophy. Ultrasonic testing (UT), acoustic emission (AE), infrared thermography (IRT), and digital shearography (DS) are commonly used, often supported by embedded sensors and machine learning algorithms for signal classification and localization [15,23,27,117,118,119,120,121,122,123,124,130]. These systems enable continuous monitoring of damage progression and support certification-driven inspection regimes mandated by the FAA and EASA [125].

In structural engineering, NDT methods are adapted for large-scale infrastructure and cost-efficiency. UT and AE are increasingly used in bridges, tunnels, and retrofitted concrete members, though their deployment is often reactive or condition-based [17,31,37,124]. Thermography and manual surveys dominate routine inspections, with drone-assisted IRT and semi-automated scanning systems emerging as scalable solutions for field deployment [31,37]. AE is particularly valuable for monitoring bond degradation and creep-related failures in FRP-strengthened and retrofitted concrete structures [17,18,37,60]. However, embedded SHM systems remain rare due to cost, complexity, and limited standardization, despite their demonstrated potential for long-term monitoring of delamination growth and interface deterioration [126].

To synthesize sector-specific applications, Table 8 presents a comparative overview of NDT and SHM techniques used in aviation and structural engineering, based on literature sources [15,17,18,23,26,27,31,35,37,117,118,120,122,123,124,129,130,131,132]. While aviation prioritizes precision, certification compliance, and embedded monitoring, structural engineering emphasizes scalability, cost efficiency, and adaptability for large-scale infrastructure. This divergence reflects the broader contrast in safety philosophies discussed in Section 3.3: aviation adopts a damage-tolerant approach supported by proactive inspection, while structural engineering relies on durability-based design and periodic condition assessment.

These distinctions underscore the need for sector-specific adaptation of inspection technologies and highlight opportunities for cross-sectoral integration. For instance, the embedded SHM systems and machine learning-enhanced AE classification used in aerospace could inform the development of scalable monitoring frameworks for critical infrastructure. Conversely, the pragmatic deployment strategies in civil engineering may offer insights into cost-effective inspection planning for non-critical aerospace components.

### 4.3. Inspection Regimes and Operational Integration

Sector-specific safety philosophies and operational constraints shape inspection regimes for FRP laminates. In aviation, inspections are preventive and scheduled, mandated by certification authorities such as the FAA and EASA. Techniques like phased-array UT and DS are used to assess bonded joints and composite skins, while embedded AE and fiber optic sensors provide continuous surveillance [117,118,121,127].

Structural engineering, in contrast, relies on condition-based or reactive inspections triggered by visible damage or performance anomalies. Manual techniques, such as hammer sounding and visual surveys, remain common, although AE and thermography are increasingly adopted for critical infrastructure [31,37,124,132]. Embedded monitoring remains rare due to its high cost and limited scale, but long-term studies demonstrate its potential for detecting delamination growth and interface degradation. These inspection regimes complement the comparative overview of techniques presented in Table 8, which maps operational deployment across sectors.

These divergent inspection philosophies reflect the underlying design logic of each sector. Aviation integrates inspection into a damage-tolerant framework, ensuring that structures remain safe despite the presence of flaws. Structural engineering embeds durability into the design, thereby minimizing the need for frequent inspections and maintenance. The integration of machine learning and SHM technologies offers a pathway toward harmonizing these approaches, enabling scalable, predictive maintenance across domains. These operational insights lay the groundwork for Section 5, which examines how predictive modeling frameworks simulate degradation and inform certification or design decisions across various sectors.

## 5. Predictive Modeling and Damage Analysis

The preceding sections have examined how sector-specific service conditions and degradation mechanisms shape the design and inspection strategies of laminated FRP composites in aviation and structural engineering. Section 5 builds upon this foundation by shifting the focus from damage detection and environmental deterioration to predictive modeling and degradation assessment. In contrast to the inspection-centric approaches discussed in Section 3 and Section 4, this section examines how modeling frameworks are utilized to simulate damage evolution, assess long-term performance, and inform certification or design decisions. The comparative analysis emphasizes the depth, resolution, and regulatory integration of modeling practices across sectors, highlighting both shared analytical foundations and divergent philosophies. Particular attention is given to the calibration and validation strategies that underpin modeling fidelity, as well as emerging hybrid frameworks—such as physics-informed neural networks and machine learning surrogates—that offer new opportunities for cross-sectoral integration.

### 5.1. Shared Analytical Foundations: CLT and FEM in Sectoral Contexts

The analytical modeling of FRP laminates in both aviation and structural engineering typically begins with CLT, which provides baseline stiffness estimates and supports early-stage design. As discussed in Section 2.4 and summarized in Table 4, CLT forms a shared analytical foundation across sectors. In predictive modeling workflows, however, CLT is not used in isolation; it is extended through numerical simulations and damage modeling frameworks that reflect sector-specific performance demands [133,134].

**Figure 1:**
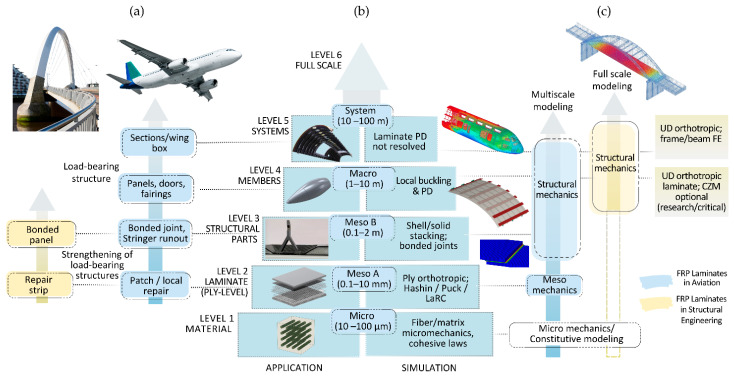
Multiscale consideration framework for FRP laminates in aviation and structural engineering: (**a**) application range; (**b**) Levels 1 to 6: alignment of simulation and validation scales; (**c**) modeling strategies—multiscale progressive damage (aviation) vs. full-scale FEM with empirical simplifications (structural engineering). Note: UD = unidirectional; PD = progressive damage.

Finite element modeling (FEM) builds upon CLT to simulate three-dimensional structural behavior, incorporating ply architecture, geometric complexity, and boundary conditions. In aviation, FEM is used to model progressive damage and fatigue degradation, while in structural engineering, it primarily supports global strength and serviceability assessments under static or quasi-static loads [52,135]. To synthesize the sector-specific modeling philosophies, Figure 1 illustrates the multiscale framework for FRP laminate simulation in aviation and structural engineering, highlighting application range, simulation depth, and validation logic. As illustrated in Figure 1, aviation modeling emphasizes multiscale simulation and progressive damage validation across building-block levels, while structural engineering relies on global FEM supported by empirical degradation logic. These modeling strategies reflect sector-specific priorities and validation pathways. Notably, the schematic does not explicitly incorporate environmental aging effects, which are introduced through distinct mechanisms in each sector.

In structural engineering, environmental aging is typically addressed through empirical reduction factors applied within FEM simulations. These factors—such as those prescribed in the technical report [78], CNR guideline [74], and *fib* Bulletin [72]—adjust tensile strength and strain limits based on exposure categories. The regulatory context for these practices is summarized in Table 4. While earlier editions of Eurocode 2 focused exclusively on traditional reinforcement, recent developments—such as the introduction of Annex R in the European standard [75]—have begun to incorporate guidance for FRP reinforcement. In contrast, aviation modeling often treats environmental degradation as a coupled or sequential modifier within fatigue and damage simulations. However, these provisions remain limited in scope and do not yet address laminate-specific environmental degradation, which continues to rely on empirical reduction approaches validated through sector-specific testing [72,84,136].

As Figure 1 depicts, aviation-level FEM incorporates ply-by-ply definitions of elastic and ultimate properties, which are calibrated using standardized mechanical tests. These include tensile (ASTM D3039), compression (ASTM D6641), and shear (ASTM D2344) procedures, as well as fatigue and delamination protocols [85,86,87]. To consolidate these sector-specific validation strategies and their supporting standards, Table 9 summarizes the calibration practices and dataset resolutions reported in the literature [18,52,53,54,55,56,72,73,74,78,85,86,87,88,89,90,135,137,138,139,140,141,142,143,144,145,146,147,148,149,150,151]. In structural engineering contexts—especially for externally bonded FRP strengthening of concrete structures—a dedicated suite of ASTM standards governs composite behavior: ASTM D7205 for tensile properties of FRP bars, ASTM D7958 for evaluating bond performance through beam testing, and ASTM D7913 for determining bar-to-concrete bond strength via pullout tests [88,89,90].

In advanced composite modeling, intralaminar failure is commonly represented through progressive damage frameworks that incorporate physically based failure criteria. Widely adopted models include the Hashin criterion [152], which distinguishes fiber and matrix failure modes; the Puck criterion [153], which emphasizes inter-fiber fracture; and the LaRC03 criterion [145], which captures non-linear shear and kink-band formation. These criteria are implemented via user-defined material subroutines—UMAT (for implicit solvers) and VUMAT (for explicit solvers)—in FEM software to simulate the initiation and evolution of fiber and matrix failure mechanisms under complex loading conditions [145,152,153]. Additionally, cohesive zone models (CZMs) are frequently integrated—often alongside the extended finite element method (XFEM)—to model interlaminar delamination within explicit FEM environments, using user subroutines for interface behavior [142,150,154]. These modeling approaches are validated through the building-block certification process mandated by FAA [53], EASA [56], and SAE International [54,55]. This process progresses from coupon-scale tests (small specimens) to element and subcomponent levels, ensuring that both failure initiation and progression are captured under representative loading and environmental conditions. The validation logic and associated standards are summarized in Table 9.

The critical distinction between aviation and structural engineering lies in the modeling of intent and depth: structural engineering primarily uses CLT/FEM for early-stage global design. It relies on empirical degradation adjustments, whereas aviation systematically extends CLT/FEM through multiscale and progressive damage frameworks to predict fatigue-related failure and residual performance across service life [18,146].

### 5.2. Divergent Modeling Philosophies and Regulatory Drivers

While foundational tools such as CLT and FEM provide a widespread analytical and computational basis for composite modeling across various engineering domains, the divergence in modeling philosophies becomes pronounced when sector-specific objectives, regulatory frameworks, and performance expectations are considered. As summarized in Table 4 and discussed in Section 2.4, these frameworks reflect distinct safety philosophies—damage tolerance in aviation and durability-based design in structural engineering (Table 1). These distinctions are particularly evident when contrasting the aviation and structural engineering sectors, where the same modeling infrastructure supports fundamentally different design imperatives.

In aviation, the FRP modeling is inextricably linked to certification requirements and the safety-critical nature of structural performance. Here, modeling extends beyond preliminary design estimation and is closely tied to damage-tolerance principles as codified in the certification standards listed in Table 4. Accordingly, modeling workflows integrate progressive damage models (PDM) to simulate intralaminar failure mechanisms—including fiber breakage, matrix cracking, and shear failure—based on stress- or strain-based criteria derived from experimentally validated input data [143,146,147,151]. The CZM is further employed to represent interlaminar delamination, a dominant failure mechanism in layered composites, often in conjunction with the XFEM. These failure mechanisms—particularly matrix cracking and delamination—were previously discussed in Section 4.2 and Table 2 as critical degradation issues in the aviation industry.

A noticeable distinction between the sectors lies in the role of predictive modeling versus SHM [155]. In aviation, predictive modeling is deeply embedded in the certification process, with high-fidelity simulations used to anticipate damage initiation and progression under defined mission profiles [156]. SHM systems, while present, serve primarily as redundancy layers to validate model predictions and ensure operational safety. This validation role of SHM contrasts with its damage detection function in structural engineering, as discussed in Section 4.2 and Table 8. In contrast, structural engineering often relies more heavily on SHM and periodic inspection to assess long-term performance, as predictive models typically lack the resolution or environmental coupling needed for accurate degradation forecasting [157]. This reliance on inspection aligns with the durability-based design logic outlined in Section 2.3 and the degradation mechanisms induced by harsh environments, as summarized in Table 2. This divergence reflects not only differences in regulatory expectations but also in the maturity of modeling frameworks and the availability of high-resolution input data. As detailed in Table 9, aviation modeling benefits from ply-level datasets and multi-scale validation, whereas structural engineering relies on member-level performance metrics and empirical reduction factors.

XFEM improves traditional FEM by allowing discontinuities—such as cracks and delamination—to propagate within elements independently of the underlying mesh topology. By enriching the solution space with special functions, XFEM can capture crack initiation and growth without the need for continual mesh refinement or remeshing, thereby significantly improving computational efficiency in modeling fracture phenomena. In composite simulations, XFEM is particularly effective for tracking delamination fronts and matrix cracks, and is often combined with interface elements or cohesive laws to simulate complex damage interactions at ply interfaces and free edges [144,148,149].

Furthermore, aviation modeling explicitly accounts for fatigue degradation, given the sector’s focus on long-term operational reliability under cyclic loading. Empirical S-N curves are combined with damage accumulation laws (e.g., Miner’s rule, strain-life models) and implemented via user-defined material subroutines to simulate stiffness loss, strength reduction, and damage progression over the service life. These predictive capabilities are validated through a building-block approach. They are crucial for airworthiness certification, which requires not only precise failure load predictions but also the modeling of damage progression under expected mission profiles [52,133,134].

In contrast, structural engineering prioritizes durability estimation and long-term service performance under diverse environmental conditions rather than immediate failure prediction. While FEM remains a core tool, it is primarily used to evaluate structural adequacy under static and quasi-static loading, with damage typically modeled implicitly. Instead, empirical aging and degradation models are employed, wherein environmental reduction factors—derived from accelerated conditioning tests—adjust material properties to account for effects such as moisture ingress, UV exposure, and thermal cycling. For example, service life estimations for externally bonded FRP laminates in bridge strengthening projects are conducted using exposure-specific strain reduction coefficients, as prescribed in standards and guidelines [74,78,135]. These coefficients are embedded within FEM simulations through modified elastic properties or strength limits, allowing engineers to satisfy safety and serviceability criteria defined by structural engineering codes, such as the *fib* Bulletin [72], Eurocode 2 [75], and ACI Committee 440 guideline [73].

Therefore, the essential divergence lies in the role that modeling plays within each domain’s design philosophy. In aviation, modeling is a predictive, high-fidelity tool embedded in the certification pipeline and required to anticipate complex failure mechanisms under both monotonic and fatigue loading. In contrast, in structural engineering, modeling serves primarily as a conservative estimator of structural capacity and long-term performance, constrained by empirical degradation factors rather than physically based damage mechanics. This distinction reflects not only differences in safety margins and service expectations but also the underlying regulatory and operational frameworks that shape how composite modeling is practiced across sectors.

### 5.3. Calibration Logic and Hybrid Modeling Frameworks

As discussed in Section 2.1, Section 2.2, Section 2.3, Section 3.3, and Section 5.1, aviation modeling is embedded within certification workflows governed by FAA and EASA regulations [53,54,55,56], which require simulating progressive failure mechanisms—such as matrix cracking, delamination, and fiber breakage—under cyclic and impact loading [137,138,139,140,141]. These mechanisms are addressed through multiscale simulations and progressive damage models, calibrated using ply-level data and validated through building-block testing, as detailed in Table 9. In contrast, structural engineering depends on long-term in-service monitoring, accelerated aging tests, and staged proof-load trials [18,72,74,78,88,89,90], with models typically operating at the member level and incorporating empirical degradation factors. These calibration contrasts form the basis for hybrid modeling frameworks, which aim to integrate multiscale simulation logic with data-driven inference to address complex degradation mechanisms across sectors.

The development of hybrid composite systems—particularly those combining glass and carbon fibers [62,63,64,65,66]—introduces additional modeling complexity due to fiber–matrix interactions, differential aging behavior, and anisotropic damage evolution. These systems require hybrid modeling approaches that integrate multiscale simulation logic with data-driven inference, enabling accurate prediction of performance under diverse service conditions.

Bridging this gap requires modeling frameworks that can integrate physical degradation mechanisms with data-driven inference. Recent developments in computational mechanics and artificial intelligence have introduced promising tools for this purpose. Physics-informed neural networks (PINNs), for example, embed governing equations into neural architectures, enabling efficient simulation of complex behaviors such as laminate bending, fatigue progression, or environmental aging. These models have demonstrated high accuracy in replicating finite element results while significantly reducing computational cost [158,159]. Similarly, hierarchical physically recurrent neural networks (HPRNNs) have been proposed for woven composites, offering microstructure-level surrogate models that generalize well across various loading scenarios [160].

In both sectors, ML-assisted surrogate models are being increasingly used to accelerate fatigue life prediction, optimize stacking sequences, or estimate residual strength under various conditions [161,162]. These tools are especially useful in structural engineering, where long-term monitoring data can be utilized to train predictive models that account for site-specific exposure histories. On the other hand, aviation can gain from including environmental degradation logic—such as moisture diffusion or UV-induced embrittlement—into its fatigue and impact simulations, as discussed in Section 3.2.

The comparative insights developed throughout Section 5 are consolidated in Table 10, which summarizes the modeling tools, failure criteria, and validation strategies employed in each sector. This synthesis highlights not only the divergence in modeling depth and regulatory integration but also the shared analytical foundations—such as CLT and FEM—that can support hybrid approaches. By combining physics-based models with ML surrogates—such as physics-informed neural networks (PINNs) and hierarchical physically recurrent neural networks (HPRNNs)—it becomes possible to construct scalable, interpretable, and computationally efficient frameworks [160,163].

The last column in Table 10 introduces a qualitative indicator of integration potential, reflecting the feasibility of transferring modeling practices and analytical tools between aviation and structural engineering. This classification—categorized as “High”, “Moderate”, or “Low”—is not prescriptive but rather illustrative, intended to guide readers toward identifying areas of methodological convergence. The integration of modeling strategies is particularly relevant in the context of hybrid frameworks, which aim to combine the predictive fidelity of aerospace simulations with the scalability and environmental realism of infrastructure-oriented models. As discussed by Diniță et al. [6] and Agarwal et al. [164], such frameworks benefit from shared analytical foundations (e.g., CLT, FEM) and emerging machine learning techniques (e.g., PINNs, HPRNNs). Still, sector-specific validation protocols and data resolution disparities constrain their adoption. The integration potential thus reflects not only technical compatibility but also regulatory flexibility, dataset availability, and computational scalability. By identifying transferable methodologies, this classification supports the broader objective of developing unified modeling and inspection practices for laminated FRP composites across engineering domains.

Despite the modeling advances listed in Table 10, several challenges remain. The training of PINNs and other hybrid models requires high-quality, standardized datasets, which are often unavailable in structural engineering. Moreover, the coupling of environmental and mechanical degradation remains limited in both sectors, as discussed in Section 5.3. Addressing these limitations will require closer integration between experimental testing, numerical modeling, and structural health monitoring, as well as the development of shared validation protocols across industries.

These considerations set the stage for the broader discussion in the following section. Section 6 synthesizes the comparative findings of the review, evaluates the versatility of modeling and inspection techniques across sectors, and identifies future research directions aimed at unifying predictive frameworks for laminated FRP composites.

## 6. Comparative Outlook and Verified Overview

This section synthesizes the comparative insights developed in this literature survey, evaluating their implications for cross-sectoral integration, methodological advancement, and future research. The discussion focuses on how sector-specific modeling depth, inspection strategies, and environmental degradation logic shape the performance and reliability of laminated FRP composites. It also verifies whether the conclusions formulated in this review are aligned with existing literature and identifies opportunities for knowledge transfer and hybrid framework development that could unify predictive methodologies across aviation and structural engineering.

### 6.1. Modeling Depth and Scalability: Bridging Fidelity and Feasibility

As illustrated in Section 5.3, the modeling depth and calibration logic diverge significantly between aviation and structural engineering. To exemplify this contrast, two representative configurations can be considered. In aviation, a fuselage skin panel with stringer-stiffened architecture is subject to barely visible impact damage (BVID), typically detected via ultrasonic C-scan and managed through scheduled non-destructive inspections (NDIs) aligned with certification protocols. Residual compression-after-impact (CAI) strength is evaluated as a percentage of original performance, with values ranging from 40% to 70% [55], depending on laminate architecture and toughening strategies. In structural engineering, an externally bonded CFRP plate applied to a reinforced concrete member is designed for a specific exposure class (e.g., UV and moisture), with environmental reduction factors applied to tensile strength and strain limits. Typical reduction coefficients range from 0.85 to 0.50 for strain limits [74] and about 0.51 for tensile strength [78], depending on exposure severity. Long-term performance is monitored through periodic inspection or acoustic emission (AE), using stiffness loss and deflection trends to evaluate serviceability. These examples reinforce the modeling and inspection philosophies discussed earlier: damage tolerance in aviation and durability-based design in civil infrastructure.

The conclusion that aviation modeling achieves higher fidelity, while structural engineering emphasizes feasibility, is strongly supported by recent publications. Zhang et al. [165] demonstrated that progressive damage modeling using Hashin and Puck criteria accurately captures compression-after-impact behavior in aerospace laminates, validating the fidelity of aviation simulations. Similarly, Yan et al. [166] confirmed the effectiveness of embedded fiber Bragg grating (FBG) sensors for real-time monitoring of thermal and mechanical degradation, reinforcing the integration of high-resolution data into aviation modeling workflows.

In contrast, Hussnain et al. [18] and Harle [60] emphasize the reliance on empirical degradation models and long-term durability assessments in structural engineering, consistent with the simplified modeling logic discussed in Section 2.3 and Section 5.2. These studies confirm that structural engineering models often lack ply-level resolution and instead depend on exposure-specific reduction coefficients, as summarized in Table 9.

The feasibility of hybrid modeling frameworks—combining aviation-grade fidelity with structural-scale applicability—is supported by Diniță et al. [6], who advocate for AI-driven simulation tools that integrate physics-based and data-driven approaches. Agarwal et al. [164] and Azanaw [167] further substantiate this direction by reviewing surrogate modeling strategies and highlighting the potential of physics-informed neural networks (PINNs) to bridge the fidelity-feasibility gap.

Thus, the conclusion that hybrid modeling frameworks offer a viable path forward is substantiated by recent literature. The integration of multiscale simulation logic from aviation with empirical degradation modeling from structural engineering is not only feasible but increasingly recognized as necessary for advancing service life prediction and certification across sectors.

### 6.2. Surrogate Modeling and Environmental Coupling: Promise and Limitations

The conclusion that the surrogate modeling offers a scalable alternative to high-fidelity simulations is partially supported by recent research, although limitations remain. As discussed in Section 5.3, surrogate models—particularly physics-informed neural networks (PINNs) and hierarchical physically recurrent neural networks (HPRNNs)—have demonstrated potential for accelerating fatigue life prediction and environmental degradation assessment. Diniță et al. [6] and Agarwal et al. [164] confirm that hybrid frameworks, which combine physics-based constraints with machine learning, can improve computational efficiency while preserving interpretability.

Still, the literature also highlights critical limitations. Azanaw [167] compares surrogate types and identifies trade-offs between inference speed and generalizability. While deep neural networks (DNNs) offer near-real-time prediction, they require extensive training datasets and struggle with extrapolation. Gaussian processes offer uncertainty quantification, but they scale poorly with increasing data volume, and ensemble methods often lack smoothness for gradient-based optimization. These findings reinforce the caution expressed in Section 5.3 regarding the limited availability of standardized datasets and the challenges of validating surrogate models under realistic service conditions.

To address these limitations, several initiatives have emerged aiming to standardize composite data and benchmarking protocols. The SAE International guideline [54,55] provides a foundational framework for material characterization and model validation in aerospace applications, while the *fib* Bulletin [72] and ACI Committee 440 guideline [73] offer structured test methods for FRP systems in civil infrastructure. More recently, efforts such as the American Society for Composites (ASC) surrogate modeling benchmarks [161] and physics-informed neural operator frameworks [158] have proposed reproducible datasets and validation pipelines for hybrid modeling. These resources support the development of scalable, interpretable, and certifiable surrogate models, reinforcing the feasibility of cross-sectoral integration and accelerating the adoption of hybrid frameworks in both aviation and structural engineering.

The challenge of incorporating environmental degradation into mechanical simulations, particularly in structural engineering, is widely recognized. Lin and Wang [168] highlight the complexity of multiscale degradation modeling and the essence of validated parameter transfer across different scales. Their study of interface deterioration mechanisms in FRP composites supports the idea that purely data-driven methods are insufficient for capturing coupled thermo-hygro-mechanical effects. This finding aligns with the limitations noted in Section 3.2 and Table 10, where environmental stressors are generally modeled using empirical modifiers rather than fully coupled multi-physics algorithms.

Although surrogate modeling shows promise, its current application is limited by data shortages, validation issues, and inadequate environmental integration. The idea that hybrid frameworks can balance accuracy and practicality is cautiously supported, as long as future research addresses these challenges through standardized benchmarks, reproducible protocols, and better integration with experimental and SHM data. Future work should focus on developing scalable, multi-physics algorithms and establishing shared validation standards. These developments would lead to more accurate simulation of degradation processes and promote consistency in modeling practices across the considered sectors.

### 6.3. Validation and Standardization: Sectoral Bottlenecks and Integration Potential

Recent publications strongly support the conclusion that validation and standardization remain critical bottlenecks across sectors. As discussed in Section 4.2 and Section 5.3, aviation modeling benefits from rigorous certification protocols and building-block validation strategies, while structural engineering relies on empirical degradation models and in-service monitoring. This divergence is reflected in the calibration practices summarized in Table 9 and the inspection strategies outlined in Table 8.

Zhang et al. [165] and Yan et al. [166] emphasize the importance of validating progressive damage simulations against experimental data, particularly in aviation contexts. Their studies confirm that embedded sensors and high-resolution monitoring systems can enhance model fidelity and support certification workflows. These findings align with the damage-tolerant philosophy discussed in Section 3.3 and the predictive modeling logic outlined in Section 5.2.

In structural engineering, the lack of standardized validation protocols and benchmark datasets is a recurring concern. Agarwal et al. [164] and Azanaw [167] both highlight the absence of reproducibility standards in ML-based damage assessment tools, noting that many published models lack accessible code, consistent evaluation metrics, or cross-domain applicability. This limitation is consistent with the challenges described in Section 5.3, where empirical degradation models are often calibrated using project-specific data, limiting generalizability.

Despite these limitations, the literature supports the idea that cross-sectoral integration is possible. Ogunleye et al. [155] and Yan et al. [166] show that sensor fusion and AI-enhanced SHM frameworks created for aerospace uses can be adapted for monitoring civil infrastructure. On the other hand, practical deployment methods in structural engineering—such as drone-assisted thermography and semi-automated inspections—may guide cost-effective maintenance planning for non-critical aerospace parts.

In summary, the conclusion that validation and standardization are shared bottlenecks is fully aligned with existing literature. Addressing these challenges through unified protocols, shared datasets, and cross-sectoral benchmarking will be essential for harmonizing modeling and inspection methodologies for laminated FRP composites.

As detailed in Section 5, aviation modeling achieves high fidelity through multiscale simulations and embedded monitoring, while structural engineering emphasizes scalable design and empirical degradation logic. Surrogate modeling and hybrid frameworks present promising opportunities for integration, although their adoption remains limited by validation and data constraints. Insights into sectoral differences in modeling depth, inspection strategies, and environmental interaction highlight the need for unified approaches that balance fidelity, scalability, and realistic environmental representation. These considerations lay the groundwork for the broader discussion in the next section, which summarizes the key findings of this review and outlines future research directions aimed at enhancing the performance and reliability of laminated FRP composites in the two industry sectors examined.

## 7. Conclusions and Future Perspectives

This comparative review has systematically compared the application of laminated FRP composites in aviation and structural engineering, revealing sector-specific divergences in design logic, degradation mechanisms, modeling depth, and inspection strategies. The comparative analysis conducted in Section 6 confirms that the conclusions formulated throughout the manuscript are broadly supported by recent research.

Aviation engineering uses high-fidelity modeling techniques, supported by ply-level datasets, damage simulations, and embedded monitoring systems. These methods are closely tied to certification processes, allowing for predictive assessments of fatigue, impact, and environmental wear. In contrast, structural engineering emphasizes scalable design and long-term durability, employing member-level simulations and empirical degradation models calibrated through accelerated aging tests and field observations. Surrogate modeling and hybrid frameworks—such as physics-informed neural networks—offer promising paths for bridging the gap between fidelity and feasibility across these sectors. Although data availability and validation protocols currently limit their use, recent advances suggest that scalable, interpretable, and physically grounded models can enhance service life predictions across various fields. Incorporating environmental degradation into mechanical simulations remains a significant challenge, particularly in structural engineering; however, hybrid approaches offer a practical solution to integrating environmental degradation into mechanical simulations.

Validation and standardization are common bottlenecks. Aviation benefits from strict certification processes and multi-level testing, while structural engineering encounters challenges due to fragmented guidelines and project-specific adjustments. However, both fields are moving toward unified modeling and inspection methods, aided by advances in sensor technology, ML, and cross-sector benchmarking.

### 7.1. Novelty of Review

Unlike previous publications that focus exclusively on either aviation or structural engineering applications of FRP composites, this review provides a structured, literature-supported comparison of laminated FRP systems across both sectors. The novelty lies in the synthesis of modeling depth, inspection strategy, and environmental degradation logic, framed through a sectoral lens and verified against recent review-level sources.

Each conclusion is substantiated not only by technical analysis but also by explicit alignment with the existing literature, ensuring that the comparative insights are both original and grounded in established research. The manuscript introduces a hybrid perspective that integrates the precision of aviation modeling with the durability logic of structural engineering. This integrative approach has not yet been consolidated in the available literature.

### 7.2. Future Development Perspectives

The future of FRP composite engineering depends on merging strengths from different sectors. Aviation can strengthen structural engineering by sharing high-fidelity modeling practices, progressive damage frameworks, and embedded SHM systems for infrastructure. These tools may enhance the predictive fidelity of structural models and support the development of scalable monitoring frameworks. In contrast, structural engineering provides aviation with valuable insights into environmental durability, exposure classification, and life-cycle cost optimization. The practical inspection methods and empirical degradation models used in civil infrastructure can inform the development of robust and cost-efficient maintenance protocols for non-critical aerospace parts. To promote cross-sector collaboration, future research could focus on the following areas.

- Standardized datasets capturing coupled environmental–mechanical degradation are essential for advancing predictive modeling. The development of open-access repositories and reproducible benchmarks may facilitate cross-sectoral validation and support the integration of hybrid frameworks.
- The incorporation of SHM data into modeling workflows offers a promising avenue for adaptive simulation. Real-time sensor feedback—particularly from embedded systems—could enhance the fidelity of damage progression models and inform maintenance strategies.
- Hybrid modeling frameworks that combine physics-based and data-driven approaches merit further exploration. Techniques such as PINNs and HPRNNs, trained on certified datasets, may offer scalable and interpretable solutions for simulating fatigue, delamination, and environmental aging.
- The development of hybrid reinforcement systems—such as glass–carbon fiber laminates—offers significant potential for maximizing mechanical performance while minimizing material consumption and structural weight. These systems warrant further investigation through hybrid modeling frameworks that can capture their complex degradation and interaction mechanisms.
- While aligning building-block testing procedures and exposure classification standards may enhance methodological compatibility, complete unification of certification strategies across sectors remains unlikely due to fundamentally different serviceability and resistance requirements. Nevertheless, comparative analysis of failure mechanisms and validation practices can support knowledge transfer and inspire improvements in sector-specific methodologies.

Along with methodological improvements, future progress might also be aided by educational initiatives that encourage cross-sector understanding. Incorporating aviation-grade modeling techniques and infrastructure-focused durability logic into graduate programs could enhance interdisciplinary skills among new researchers. Combined training courses, shared simulation tools, and joint doctoral programs can help develop common expertise in composite design, inspection, and service life estimation, ultimately strengthening the integration of engineering approaches across the analyzed sectors.

The comparative analysis presented in this review demonstrates that aviation and structural engineering represent two distinct yet complementary paradigms in the application of laminated FRP composites. Aviation contributes high-fidelity modeling, progressive damage simulation, and embedded monitoring systems, while structural engineering offers scalable inspection strategies, exposure-based degradation logic, and long-term durability frameworks. These sector-specific strengths are not mutually exclusive; instead, they provide synergistic opportunities for cross-sectoral integration. By bridging the modeling precision of aviation with the environmental realism and scalability of structural engineering, future research can develop unified, sustainable, and adaptive engineering practices. Such integration may foster the development of FRP composite technologies that are certifiable, resilient, environmentally robust, and economically viable across diverse engineering domains.

## Figures and Tables

**Table 1:** Sectoral comparison of FRP laminates in aviation and structural engineering.

Aspect	Aviation Engineering	Structural Engineering
Primary design philosophy	Damage tolerance: failure is expected, but must remain controlled and predictable	Durability-based: failure is avoided through conservative design and safety margins
Typical loading conditions	Cyclic, vibratory, and impact loads; fatigue-critical	Static or sustained loads and environmental aging dominate
Laminate configuration	Thin-ply, optimized stacking sequences for weight and fatigue resistance	Standard layups, pultruded profiles briefly used, durability-focused architecture
Failure mechanisms	Delamination, matrix cracking, fiber breakage; barely visible impact damage (BVID)	Environmental degradation, interface debonding, visible cracking
Inspection strategy	Scheduled NDT and embedded SHM systems; certification-driven	Reactive or periodic inspection; manual or semi-automated methods
Modeling approach	Multiscale FEM, progressive damage models, cohesive zone modeling, fatigue simulation	CLT/FEM for global response; empirical degradation factors for aging effects
Regulatory framework *	FAA, EASA, SAE International; certification mandates predictive modeling	Eurocode 2, *fib* Bulletins, and ACI Committee 440 and CNR guidelines; design codes emphasize serviceability and safety
Data resolution	Ply-level mechanical and fatigue data, validated through building-block testing	Member-level performance metrics; empirical reduction coefficients
Research focus	High-fidelity modeling, residual strength, fatigue life prediction	Long-term durability, bond retention, and environmental exposure effects

* FAA = Federal Aviation Administration; EASA = European Aviation Safety Agency; SAE International = Society of Automotive Engineers (former name); *fib* = International Federation for Structural Concrete; ACI = American Concrete Institute; CNR = National Research Council (Italy).

**Table 2:** * Sector-specific failure causes, degradation mechanisms, and detection methods in laminated FRP composites.

Failure Type	Aviation Engineering	Structural Engineering
Matrix cracking	Stress concentration accelerates delamination (early-stage damage) [15,27]	Surface whitening or cracking and visible degradation [17,35,36]
	AE, UT	Visual inspection, thermography
Fiber–matrix debonding	Load transfer reduction and interlaminar failure initiation [23,27]	Interface deterioration accelerated by moisture/UV [17,18,37]
	AE, UT	Thermography, AE
Delamination	CAI strength reduction propagated under fatigue (critical failure) [15,26,27,30]	Stiffness reduction [17,31,37]
	AE, UT, shearography	AE, UT, thermography
Fiber breakage/kinking ^‡^	Catastrophic loss of strength (final failure stage) [21,22,23,27]	Failure that precedes matrix/interface degradation [18,37]
	AE, UT	AE, long-term monitoring
Creep/fatigue effects	Fiber failure accelerated by delamination under fatigue [21,24,25,28,30]	Stiffness reduction and deformation increase under sustained load [17,18,37]
	AE, UT	AE, long-term monitoring
Environmental degradation	Matrix and interface degradation under thermal cycling, humidity, and de-icing [20,27,29]	Long-term bond degradation under UV, freezing–thawing cycles, and moisture ingress [18,19,37]
	AE, thermography	AE, thermography, visual inspection
External bond failure	–	Failure of strengthening systems; reduction in flexural/shear capacity [17,31,32,33,36,37]
	–	UT, thermography, visual inspection
Interlaminar shear failure	–	Loss of laminate integrity; stress concentrations at ply interfaces [7,17,31]
	–	AE, UT

* AE = acoustic emission; UT = ultrasonic testing; CAI = compression-after-impact; UV = ultraviolet. ^‡^ Fiber kinking is a prevalent compressive failure in aligned-fiber laminates, prominent in aerospace laminates, but also appears locally in compressive zones and anchor regions of building structures.

**Table 3:** * Typical thin-ply product forms and performance trends.

Form	Thickness	Delamination	Fatigue ^‡^	CAI	Resistance Mechanism
UD prepreg	≤100 µm	↑ onset	↑ life	↑	Delayed matrix-crack onset; reduced interlaminar driving forces [13,45,48]
Spread-tow/ thin woven	Near 60–100 µm	↑	↑ life	↑	Reduced tows smooth stress fields; arrested cracks [13,44,47]
Hybrid thin/ thick stacks	60–100 µm (thin ply)	↑	↑ life	↑	Thin plies arrest cracks; thick plies carry the load [14,29]
Gradient thickness stacks	Graded near 50–150 µm	↑ impact tolerance	Context- dependent	↑	Reduced interlaminar stress peaks [29,43]
Ultra-thin laminates	Typically, below 60–80 µm	↑	↑ life	↑	Diminished micro-damages; crack confinement [15,43]

* UD = unidirectional; CAI = compression after impact; ↑ = enhancement regarding the conventional 125–200 µm thickness plies (these trends are qualitative but not design-enabled). ^‡^ life = longer life at a given stress rate (qualitative trend synthesis from cited studies).

**Table 4:** * Sector-specific regulatory frameworks and design standards for FRP composites.

Regulation	Aviation Engineering	Structural Engineering
Standards	FAA, EASA	CEN, ISO, ASTM International
	Composite Aircraft Structure AC 20-107B; Rules for Large Aeroplanes CS-25.603–25.613 [53,56]	Eurocode 2 EN 1992-1-1:2023; ISO 527-4:2023; ASTM Standard Test Methods [75,84,85,86,87,88,89,90]
Guidelines	SAE International	*fib*, Concrete Society, CNR, ACI Committee 440
	Composite Materials Handbook [54,55]	*fib* Model Code 2020; *fib* Bulletins; Design Guides TR 55, CNR-DT 200 R1/2013, and ACI PRC-440.2-23; Guide Test Methods ACI PRC-440.3-12 [72,73,74,76,78,83]
Technical reports	NASA	JRC, TRB
	Reports NASA/TM-97-206317, NASA/TM-2005-213530 [79,80]	JRC Report EUR 27666 EN; NCHRP Report 69 [81,82]

* FAA = Federal Aviation Administration; EASA = European Union Aviation Safety Agency; CEN = European Committee for Standardization; ISO = International Organization for Standardization; ASTM International = American Society for Testing and Materials (former name); SAE International = Society of Automotive Engineers (former name); *fib* = International Federation for Structural Concrete; CNR = National Research Council (Italy); ACI = American Concrete Institute; NASA = National Aeronautics and Space Administration (USA); JRC = Joint Research Centre (Italy); TRB = Transportation Research Board (USA).

**Table 5:** Sectoral comparison of degradation mechanisms and damage detection practices.

Aspect	Aviation Engineering	Structural Engineering
Exploitation duration	Typically, 20–40 years; governed by fatigue-critical mission profiles [26,27,30]	Often exceeds 50 years; governed by environmental durability and structural redundancy [35,37,119]
Service environment	Controlled but mechanically aggressive: cyclic loads, vibration, impact, temperature [27,30,39]	Harsh and variable: moisture, UV radiation, freezing–thawing cycles, chemical exposure [35,37,119]
Dominant degradation mechanisms	Matrix cracking, fiber breakage, delamination, BVID [15,23,27,30,39,48,49]	Moisture ingress, UV-induced resin degradation, interfacial debonding, and creep aging [31,35,37,60,119,126]
Design philosophy	Damage-tolerant: failure initiation is expected and must be predictable and non-critical [26,27,30,38]	Durability-based: conservative design and empirical factors prevent failure [6,35,37,60]
Damage detection strategy	Embedded SHM systems, scheduled NDT (e.g., UT, AE, thermography), certification-driven [21,22,23,24,117,121,125]	Periodic inspection, semi-automated NDT, and reactive maintenance [17,18,31,33,36,37,124]
Detection resolution	High-resolution, ply-level monitoring; fatigue and impact damage tracking [23,120,122,123]	Member-level or surface-level detection, focused on visible or environmental damage [31,35,37,119]
Inspection frequency	Regular intervals defined by certification protocols (FAA, EASA) [53,56,117,118,125]	Project-specific, often less frequent, and condition-based [31,37,76,124]

**Table 6:** * Representative sector-dependent quantitative indicators of FRP laminates.

Parameter	Aviation Engineering	Structural Engineering
Materials	Carbon fiber reinforcement + epoxy matrix	Glass or carbon fiber reinforcement + epoxy matrix
Knock-down factors	Hot/wet environment or fatigue [54]: ↓ stiffness by 10% to 30% ↓ strength by 20% to 50% (system-dependent)	Hot/wet environment or fatigue [60,72,73]: ↓ stiffness by 15% to 40% ↓ strength by 30% to 60% (exposure-dependent)
Residual CAI	↓ by 30% to 60% (depending on the impact energy, layup, and toughening) [2,48,55]	↓ by 40% to 65% [3,72]
Fatigue slope ^•^	From 8.3 to 20 (tension-tension load) [55]	From 6.7 to 12.5 (glass fibers; moisture-sensitive) [91]
Moisture diffusion coefficient	Room to elevated temperature: from 10^−7^ cm^2^/s to 10^−9^ cm^2^/s (matrix-dependent) [55]	Room to elevated temperature: from 10^−6^ cm^2^/s to 10^−8^ cm^2^/s (glass fibers) [7,60,65]
UV exposure	↓ strength by 10% to 25% [55]	↓ strength by 15% to 30% [60,65]
Scheduled inspections ^†^	Visual inspection after 400 FH to 800 FH; detailed NDI after 18 to 24 months [53,56]	Typical visual inspection after 1 to 5 years; NDI depends on the service conditions.
Modeling fidelity ^‡^	**High** (progressive damage, CZM, fatigue, coupled aging; building-block validation)	**Medium** (CLT/FEM with environmental reduction factors; phenomenological fatigue)

* CZM = cohesive zone model; CLT = classical laminate theory; CAI = compression after impact; FH = flight hours; NDI = non-destructive inspection; ↓ = reduction regarding the intact FRP composite. ^•^ Fatigue strength (Basquin’s) exponent. ^‡^ Qualitative estimation. ^†^ Not regulatory rules (vary by program and operator; inspection philosophy and documented practices shown where available).

**Table 7:** Mapping of NDT and SHM methods to failure types in laminated FRP composites.

Method	Failure Type	Monitoring Depth	Damage Size	Use in Service	Sector Cadence
Ultrasonic testing	Delamination, debonding, BVID	Through-thickness	Meso → macro (from near 5 mm to over 10 mm)	Scheduled NDI; dock/overhaul	Standard (e.g., [56]) and owner program-based
Active thermography	Near-surface delamination, debonding	Near-surface to a few mm	Meso (a few mm)	Rapid screening	Program-based/condition-based
Shearography	Debonding, core-skin issues	Near-surface	Meso → macro	Rapid area screening	Program-based
Acoustic emission	Matrix/fiber damage progress, delamination onset	Global (source location needed)	Event detection (not for sizing)	Proof/acceptance/monitoring	Event-driven/condition-based
Guided waves (Lamb)	Delamination, debonding	Long-range	Macro → meso (tuned)	SHM/monitoring	Continuous/periodic
Embedded SHM	Strain anomalies, damage indicators	Local to instrumented path	Not for sizing	Embedded/attached SHM	Continuous

**Table 8:** Comparison of NDT and SHM techniques in aviation and structural engineering.

Technique	Aviation Engineering	Structural Engineering
Ultrasonic testing (UT)	Scheduled maintenance and repair procedures (MRO) of structural components to detect delamination and cracks [15,26,27,117,118]	Applied when defects are suspected; used in bridges and tunnels [17,31,37,132]
Acoustic emission (AE)	Real-time detection of early-stage damage; embedded sensors used for fatigue and impact monitoring [15,23,27,120,122,123,129]	Used for monitoring bond degradation and creep-related failures in infrastructure [17,18,37,124]
Infrared thermography (IRT)	Non-contact, full-field inspection of near-surface defects; used in flight-line triage and maintenance [23,117,130,131]	Drone-assisted surveys and manual scans for surface anomalies [17,35,37]
Digital shearography (DS)	Detection of subsurface delamination and adhesive debonding under stress, typical for aerospace maintenance [123]	Applied for capturing bonded joints and surface strain anomalies [31]

**Table 9:** Comparison of calibration and validation practices for FRP composite modeling.

Aspect	Aviation Engineering	Structural Engineering
Primary calibration basis	Coupon-, element-, and subcomponent-scale laboratory testing under controlled loading and environmental conditions [53,54,55,56,137,138,139,140,141]	Long-term in-service monitoring, accelerated aging tests, and proof-load trials on structures [18,72,74,88,89,90]
Standards and guidelines	Mechanical properties: ASTM D2344, ASTM D3039, ASTM D6641 [85,86,87] Fatigue: ASTM D3479 [137] Impact: ASTM D7136 [140] Delamination: ASTM D5528, ASTM D6671, ASTM D7905 [138,139,141]	FRP–concrete interface and bond: ASTM D7205, ASTM D7958, ASTM D7913 [88,89,90] Tensile & bond durability: ACI Committee 440 [73], CNR guideline [74], *fib* Bulletin [72]
Typical dataset resolution	Ply-level elastic and strength properties, fracture toughness values (Modes I, II, and mixed), impact damage tolerance parameters, fatigue S-N curves [52,145,146]	Member- or system-level stiffness, strength, bond retention factors, and degradation coefficients for various environmental exposure categories [18,72,74,78]
Validation objectives	High-fidelity replication of localized damage phenomena (fiber breakage, matrix cracking, delamination) and residual strength reduction [143,144,147,148,149,151]	Assessment of long-term service performance, structural safety margins, and compliance with environmental durability factors [72,73,74]
Key limitations	High costs and time requirements for multi-scale test campaigns; variability due to manufacturing defects; limited coupling of environmental effects [135,142,150]	Lack of ply-level data; high variability in field conditions; limited availability of long-term multi-physics datasets [18,144,151]

**Table 10:** Summary of modeling tools, failure criteria, and validation strategies.

Category	Aviation Engineering	Structural Engineering	Integration Potential
Modeling tools	CLT, FEM, PDM, CZM, XFEM, multiscale simulations [52,145,146,148,150,151]	CLT, FEM, empirical degradation models, global structural assessment tools [72,74,78,147]	**High** (CLT, FEM), **Moderate** (XFEM, CZM)
Failure criteria	LaRC03 [146], Hashin [154], Puck [155] implemented via UMAT/VUMAT	No explicit ply-level failure modeling; global reduction coefficients reflect degradation [74,78]	**Low** (LaRC03), **Moderate** (Hashin), **High** (empirical coefficients)
Fatigue modeling	Certification workflow-integrated S-N curves, Miner’s rule, strain-life models [149,150]	Rarely modeled explicitly; fatigue degradation inferred from environmental exposure categories [73,147]	**Moderate**
Environmental modeling	Limited coupling; often treated sequentially or via modifiers [143,152]	Empirical reduction coefficients applied to material properties; no multi-physics integration [72,74,147]	**High**
Validation strategy	Fatigue, impact, and delamination-validated blocks: coupon → element/component → full-scale [53,54,55,56,138,139,140,141,142]	In-service monitoring, accelerated aging, proof-load testing; long-term durability-focused validation [74,88,89,90]	**Moderate**
Data resolution	Ply-level mechanical, fracture, and fatigue properties [52,148,153]	Member-level stiffness, strength, and bond retention factors [72,74,78,147]	**Moderate**
Hybrid modeling potential	PINNs, HPRNNs, and ML surrogates used for fatigue life prediction and virtual prototyping [160,161,162,163,164]	Emerging use of ML-assisted degradation models and surrogate frameworks for service life estimation [147,164]	**High**

## Data Availability

No new data were created or analyzed in this study. Data sharing is not applicable to this article.
